# Does KRAS Play a Role in the Regulation of Colon Cancer Cells-Derived Exosomes?

**DOI:** 10.3390/biology10010058

**Published:** 2021-01-14

**Authors:** Shu-Kee Eng, Ilma Ruzni Imtiaz, Bey-Hing Goh, Long Chiau Ming, Ya-Chee Lim, Wai-Leng Lee

**Affiliations:** 1 School of Science, Monash University Malaysia, Selangor 47500, Malaysia; shukeeeng@gmail.com (S.-K.E.); ilma.ruzni.imtiaz@gmail.com (I.R.I.); 2 College of Pharmaceutical Sciences, Zhejiang University, 866 Yuhangtang Road, Hangzhou 310058, China; goh.bey.hing@monash.edu; 3 Biofunctional Molecule Exploratory Research Group (BMEX), School of Pharmacy, Monash University Malaysia, Selangor 47500, Malaysia; 4 Pengiran Anak Puteri Rashidah Sa’adatul Bolkiah Institute of Health Sciences, Universiti Brunei Darussalam, Gadong BE1410, Brunei; long.ming@ubd.edu.bn (L.C.M.); yachee.lim@ubd.edu.bn (Y.-C.L.)

**Keywords:** KRAS, farnesylthiosalicylic acid, SW480, extracellular vesicles, proteomics, ACSL4

## Abstract

Exosomes are cell-derived nanovesicles, and lately, cancer-derived exosomes have been reported to carry KRAS protein, which contributes to the malignancy of many cancers. In this study, farnesylthiosalicylic acid (FTS) was used to inhibit the activities of mutated KRAS in colon cancer SW480 cells to discover the potential link between KRAS activities and cancer-derived exosomes. We observed that FTS inhibits KRAS activity in SW480 cells, but promotes their exosome production. When the exosomal proteins of SW480 cells were profiled, a total of 435 proteins were identified with 16 of them showing significant changes (greater than or equal to two-fold) in response to FTS treatment. Protein network analysis suggests KRAS inhibition may trigger stress in the cells. In addition, a high level of acetyl-coA synthetase family member 4 protein which plays an important role in colon cancer survival was identified in the exosomes secreted by FTS-treated SW480 cells. The uptake of these exosomes suppresses the growth of some cell types, but in general exosomes from FTS-treated cells enhance the recipient cell survival when compared to that of untreated cells. Together our findings suggest that FTS may trigger stress in SW480 cells, and induce more exosomes secretion as the survival messenger to mitigate the impact of KRAS inhibition in colon cancer cells.

## 1. Introduction

KRAS is one of the most prominent oncogenes found in many cancer cell types, predominantly in colon, pancreas and lung cancers [1,2]. However, there is no direct inhibitor to oncogenic KRAS, and the search for an effective therapeutic agent is still in process [3]. To date, the latest inhibitor under study has been designed towards KRAS G12C, whereby the researchers were aiming at the ability of cytosine to form a covalent bond [4]. Previous studies found that the invasive properties of mutated KRAS-bearing colon cancer cells were highly associated with Rho family GTPases (RAS homolog), which play a significant role in altering actin organization, cell-cell interaction and cell adhesion, resulting in the development of invasive properties in the cancer cells. Members of the Rho family GTPases (RhoA, Rac1 and Cdc42) were found to be involved in the transducing of intracellular signals to modulate a wide spectrum of cellular processes, including vesicle trafficking, which is important for the biogenesis of multivesicular bodies (MVB) [5]. Endocytosis leads to the formation of endosomes, and a certain late endosomal membrane encapsulates cellular molecules and forms exosomes within MVBs, which then fuse with the plasma membrane and exosome secretion to form the extracellular matrix [6]. Exosomes, 40–150 nm, are extracellular vesicles that act as messengers for cell-to-cell communication, and increasing evidence has shed light on their function in cancer progression [7]. Colon cancer-derived exosomes with the ability to travel to distant body parts have been shown to induce metastasis [8]. Recently, colon cancer-derived exosomes were found to promote cancer cell proliferation under hypoxic conditions through the activation of the STAT3 signaling pathway and the shortening of the mitotic stage [9]. Another study found that the exosomes derived from mutated KRAS-bearing colon cancer promoted the growth of non-transformed cells bearing wild-type KRAS. Moreover, these exosomes enhanced the invasiveness of the recipient cells compared to exosomes derived from wild type KRAS-bearing colon cancer cells [10].

In line with these findings, KRAS plays a role in regulating exosome formation. However, evidence to support the link between KRAS activity and exosome production remains scarce. We hypothesized that the constitutively active form of mutated KRAS, highly associated with Rho family GTPases, promotes the production of exosomes from cancer cells, owing to the membrane relatedness and vesicular trafficking association. In this study, we employed farnesylthiosalicylic acid (FTS, Salirasib), a trans-farnesylthiosalicylic acid, to treat colon cancer cells. FTS mimics c-terminal farnesyl cysteine and competes with farnesylated RAS for membrane anchorage, and subsequently abolishes the activation of RAS signaling [11,12]. The effects of FTS-inhibited KRAS on the character, composition and uptake of exosomes were investigated in our study, to verify the link between KRAS activities and colon cancer cells-derived exosomes.

## 2. Materials and Methods

### 2.1. Cell Culture

Human colon adenoma-carcinoma cell lines HT29, HCT116 and SW480, normal lung epithelial cells Beas2B, and mouse macrophage RAW264.7 were obtained from the American Type Culture Collection (Rockville, MD, USA). These cell lines were cultured in complete Roswell Park Memorial Institute medium (RPMI 1640, Sigma, St. Louis, MO, USA) which was supplemented with 10% fetal bovine serum (FBS, Gibco, Grand Island, NY, USA) and 1% penicillin/streptomycin (Gibco, Grand Island, NY, USA). SW480 cells were treated with a sub-lethal dose of farnesylthiosalicylic acid (FTS), an inhibitor of KRAS signaling purchased from Santa Cruz Biotechnology (Santa Cruz, CA, USA). The exosomes isolated from SW480 cells with or without treatment of FTS were used in the study for extracellular vesicle characterization, protein profiling and uptake by different cell types.

### 2.2. Cell Viability Assay

A 3-(4,5-dimethylthiazol-2-yl)-2,5-diphenyltetrazolium bromide (MTT) assay was performed to measure viable adherent cell numbers, as described previously [13]. To determine the sub-lethal dose of FTS, SW480 cells were seeded in 96-well plates at a density of 1 × 10^4^ cells per well and incubated at 37 °C, 5% CO_2_. After 3 h, the cells were treated with different concentrations of FTS, and an MTT assay was performed after 24 and 48 h. For the exosome uptake experiment, the tested cells were plated in 96-well plates at a density of 1 × 10^4^ cells per well. The SW480 cells were treated with 50 μM FTS at 3 h after seeding. After 18 h, all the tested cells were treated with exosomes (0.5 μg/well) derived from SW480 cells with or without FTS treatment at 37 °C for 24 h. The supernatant was discarded followed by the addition of 100 µL of 10% MTT solution. The formazan formed in the cells was then dissolved using dimethyl sulfoxide (DMSO) after 4 h of incubation. The absorbance of each well was determined at 570 nm using a Tecan plate reader. The assay was performed in triplicates and repeated three times.

### 2.3. Detection of Exosome Formation in Colon Cancer Cells

Exosome formation in the cells was compared between three established colon cancer cell lines, HT29, HCT116 and SW480, transfected with plasmid pCT-CD63-GFP (System Bioscience, Palo Alto, CA, USA). Using flow cytometry, exosome formation was determined by the percentage of cells expressing CD63-GFP, which is a protein marker that marks the location of exosomes. On the other hand, the cells expressing CD63-GFP-tagged exosomes were stained with PKH26 membrane dye and cultured on a glass coverslip. After 24 h, the cells were fixed with 4% paraformaldehyde and examined using confocal microscopic imaging.

### 2.4. Membrane-Dye Labeling of Cells and Exosomes

A red fluorescence membrane dye, PKH26 (Sigma, St. Louis, MO, USA), was used to label colon cancer cells and exosomes for visualization purposes. As referred to in the manufacturer’s protocol with slight modifications, SW480 cells (1 × 10^7^ cells) were incubated with 2 µL PKH26 in 1 mL of diluent C for 5 min followed by the addition of 5 mL complete RPMI-1640 medium to stop the staining reaction. The stained samples were centrifuged twice at 15,000× *g* for 5 min with the addition of PBS to wash and remove unbound dye. Exosomes were stained with the same protocol mentioned above, with the phosphate buffer saline (PBS) washing step performed using a Microsep™ Advance Centrifugal Device (Pall, Port Washington, NY, USA) to concentrate the stained exosomes samples.

### 2.5. Exosome Isolation

SW480 cells were cultured in RPMI medium supplemented with exosome-free FBS for 24 h. The collected media was submitted to 10 min of low speed centrifugation at 300× *g* followed by 2000× *g* for 10 min and 16,500× *g* for 30 min. At the final stage, it was pelleted twice at 120,000× *g* for 80 min at 4 °C. The resultant exosome-containing pellets were resuspended in 100 µL of PBS and stored at −20 °C until further use. The protein content of exosome preparations was determined using Bradford assay kit (Bio-rad, Hercules, CA, USA) according to the manufacturer’s protocol.

### 2.6. Transmission Electron Microscopy

A drop of exosome suspension was placed on a fomvar carbon-coated grid for 20 min followed by PBS washing. The absorbed exosomes were fixed with 2.5% glutaraldehyde for a minute followed by three rounds of washing with ultrapure water. The grid was then negatively stained with 1% uranyl acetate for 3 min and the excess fluid was drained. The grid was completely air-dried and viewed under a transmission electron microscopy (TEM), HT7700 (Hitachi, Tokyo, Japan) at 100 kV.

### 2.7. Particle Size Analysis

The sizes of the isolated exosomes were analyzed using Nanosight NS300 (Malvern, UK). A quantity of 2 µg of exosome sample was diluted with 500 µL of PBS and was injected into the sample chamber of the machine. The exosome samples were then analyzed using NTA software 3.0 (Malvern, UK) which captures the Brownian motion of the nanoparticles and generates data for the particle size and number. The machine utilized automatic settings for the maximum jump distance, and the detection threshold for each sample was 10. A total of 3 biological replicates for control and treatment group were analyzed. The particle analysis was repeated technically 3 times for each sample.

### 2.8. Western Blot Analysis

The total cell lysates for colon cancer cells (10 µg) and exosomes (10 µg) were separated on 12% sodium dodecyl sulphate-polyacrylamide gel electrophoresis (SDS-PAGE) at 120 V for 90 min and transferred to nitrocellulose membranes. After blocking in 5% non-fat milk in tris-buffered saline with tween 20 (TBST) for 2 h, the membranes were blotted with primary antibodies at 4 °C overnight for 16 h, followed by secondary bodies at a dilution of 1:2000. The protein bands were visualized using WesternBright ECL solution (Advansta, San Jose, CA, USA) and the signals were captured using a C-digit blot scanner (LI-COR). Primary antibodies against KRAS, HSC70, CD63, CD9 and CD81, and secondary antibodies (mouse anti-rabbit and goat anti-mouse) were obtained from Santa Cruz Biotechnology (Santa Cruz, CA, USA). Primary antibodies against actin and p53 were purchased from Millipore (Burlington, VT, USA) while primary antibodies against ACSL4 were obtained from Thermo Fisher Scientific (Waltham, MA, USA). Western blotting was performed in triplicate for each tested protein.

### 2.9. Protein Lysis and Digestion

The exosomal proteins were lysed in 50 μL of 1% (*w*/*v*) Sodium Deoxycholate (SDC) (Sigma, St. Louis, MO, USA). The samples were boiled at 95 °C for 5 min and sonicated thrice at 10 s each. The protein concentration of the isolated exosomes was determined using a Bradford assay kit. The proteins were denatured with dithiothreitol (DTT, Bio-rad) to a final concentration of 10 mM and incubated for 30 min at 50 °C. The samples were then alkylated by adding chloroacetamide (CAA, Sigma, St. Louis, MO, USA) to a final concentration of 40 mM, and incubated for 20 min in the dark at room temperature. A ratio of 1:100 of trypsin was added into the samples and incubated overnight at 37 °C in an incubator shaker. Trypsin digestion was stopped by adding formic acid (Sigma, St. Louis, MO, USA) to a final concentration of 1%. The lysate was then subjected to ethyl acetate precipitation. An equal volume of 100% water-saturated ethyl acetate (Sigma, St. Louis, MO, USA) was added to the lysate and vortexed thoroughly. The lysate was centrifuged at 14,000× *g* for 5 min to separate the SDC (interphase) and peptides (aqueous phase at middle layer). The aqueous phase with peptides was transferred into a new Eppendorf tube. The ethyl acetate precipitation step was repeated and the peptides were collected again. The final collected peptides were dried in a vacuum concentrator.

### 2.10. Nano-Liquid Chromatography-Mass Spectrometry/Mass Spectrometry

A QExactive Plus 1 mass spectrometer coupled with a Dionex Ultimate 3000RSLCnano liquid chromatography (LC) system (Thermo Scientific, USA) was utilized for the analysis of peptides derived from proteins in isolated exosomes. The LC system was equipped with an analytical column (Acclaim PepMap RSLC, 75 µm × 50 cm, nanoViper, C18, 2 µm, 100A; Thermo Scientific) and a trap column (Acclaim PepMap 100, 100 µm × 2 cm, nanoViper, C18, 5 µm, 100A; Thermo Scientific).

### 2.11. Protein Identification and Network Analysis

The acquired MS/MS data were analyzed using the Andromeda search engine (implemented in MaxQuant LFQ package) against the RS Uniprot human Swissprot iRT database. The raw data files were analyzed using MaxQuant to obtain protein IDs and their respective label-free quantification values using in-house standard parameters. The data were normalized based on the assumption that the majority of proteins do not change between the different conditions. The protein FDR cutoff was fixed at 1%. Statistical analysis was performed using Perseus v.1.5.8.5 after contaminants, “only identified by site” matches and reverse sequences were filtered out. The LFQ data was converted to log_2_ scale, samples were grouped by conditions and missing values were imputed based on normal distributions after all proteins that had 2 or less valid values were eliminated. The protein fold-changes of the exosomes were calculated and their significance was determined using a two-sided T-test with error-corrected *p*-values. The interactions between the identified proteins were analyzed using STRING-DB (www.string-db.org). The analysis included functional enrichment analysis for gene ontology (GO) focused on the biological processes, molecular functions and cellular components.

### 2.12. Imaging Flow Cytometry

For the uptake of labelled exosomes, the recipient cells were seeded in a 24-well plate (3 × 10^4^ cells/well) for 24 h at 37 °C, with 5% CO_2_. For FTS treatment, SW480 cells were treated with 50 μM FTS at 3 h after seeding. PKH26-labelled exosomes were added for 24 h of incubation at 37 °C, with 5% CO_2_. The cells were harvested and washed twice with PBS before the analysis using imaging flow cytometry (ImageStream X Mark II, Merck Millipore, Jaffrey, NH, USA). A minimum of 10,000 cells was analyzed for each sample and the images were captured at 40× magnification. PKH26 fluorescence were excited at the wavelength of 488 nm. The fluorescence images were collected on channel 3 (560 nm to 595 nm) while bright field images were collected on channel 4. The data collected were then analyzed using the IDEAS software (Amnis Corporation, Washington, DC, USA).

### 2.13. Statistical Analysis

All statistical analyses were performed using t-test when comparing only 2 groups and one way ANOVA with Tukey’s post hoc (*p* < 0.05) was used when 3 or more groups were involved. The experimental data were presented as mean ± standard deviation (SD). All data were analyzed using GraphPad Prism 6 software (GraphPad Software Inc., San Diego, CA, USA).

## 3. Results

### 3.1. Detection of Exosome Formation in Colon Cancer Cells

**Figure 1:**
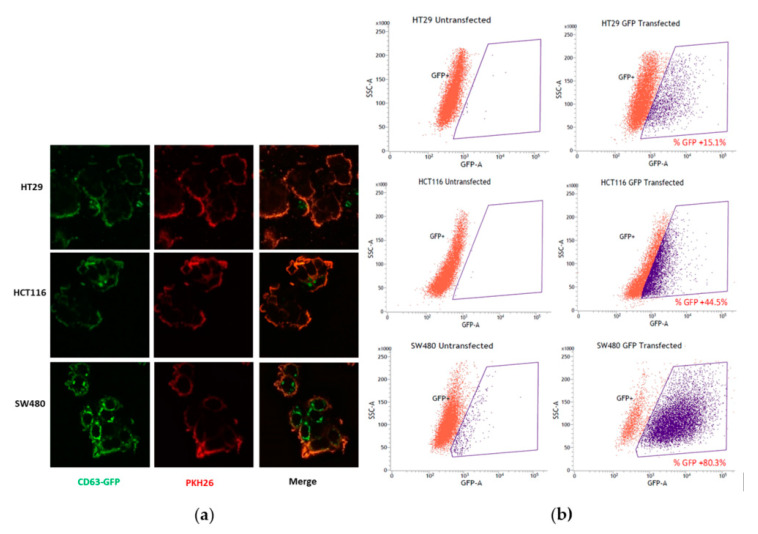
Expression of CD63-GFP in colon cancer cell lines. (**a**) Confocal microscopy; (**b**) Flow cytometric analysis.

To compare the exosome formation in colon cancer cell lines expressing different mutation statuses, three colon cell lines, HT29 (wild type KRAS), SW480 (KRAS G12V mutation) and HCT116 (KRAS G13D mutation), were transfected with plasmid pCT-CD63-GFP. Exosome production was determined by the percentage of cells expressing CD63-GFP, which is a protein marker that marks the location of exosomes. The confocal images (Figure 1a) showed the significant expression of CD63-GFP protein (green) in all three cell lines. As shown in (Figure 1b), the SW480 cells (~80%) expressed the highest level of detectable exosome protein marker CD63-GFP, as compared to the levels expressed in the HT29 (~15%) and HCT116 cells (45%).

### 3.2. Inhibition of KRAS Signaling in SW480 Cells

**Figure 2:**
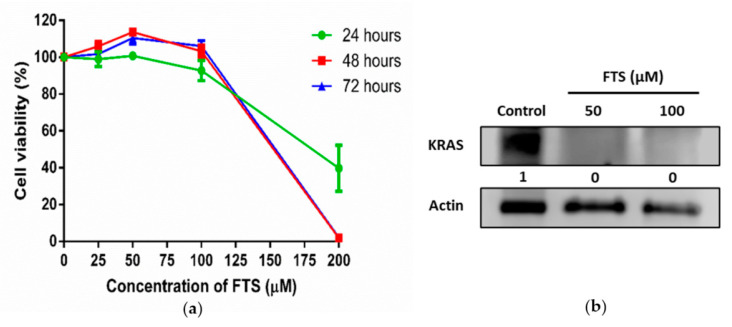
Effect of FTS treatment on cell viability and KRAS expression of SW480 cells. (**a**) Cell viability (%) of SW480 cells treated with various doses of FTS for 24, 48 and 72 h (*n* = 3); (**b**) Western blot analysis of KRAS expression in FTS-treated SW480 cells. Actin was used as loading control (*n* = 3).

SW480 cells bearing KRAS G12V mutation were treated with FTS, an inhibitor of RAS signaling in treated cells. The MTT assay of SW480 in the presence of FTS showed that the drug had induced a dose-dependent decrease in cell viability, with IC_50_ values of 180 µM, 153 µM and 155 µM for the 24, 48 and 72 h treatments, respectively (Figure 2a). Western blot analysis revealed that KRAS expression was significantly inhibited or reduced by FTS treatment, as the band showing KRAS protein (21 kDa) was only present in SW480 cells without treatment (Figure 2b, Figure S1). The presence of 50 µM FTS did not alter the morphology of SW480 cells (data not shown). Taken together, the dosage of FTS at 50 µM was sufficient to inhibit KRAS expression without affecting the growth and morphology of the treated cells.

### 3.3. Isolation and Characterization of Exosomes

**Figure 3:**
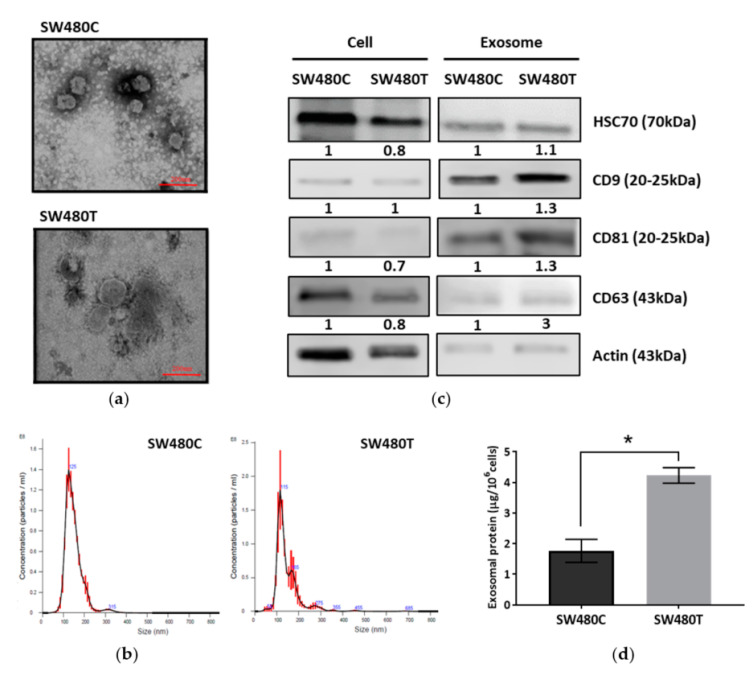
Characterization of exosomes derived from SW480 cells (SW480C—Control cells; SW480T—FTS-treated cells). (**a**) Transmission electron micrographs; (**b**) Particle size analysis by Nanosight; (**c**) Western blot analysis of exosomal markers with actin as the loading control; (**d**) Exosome production analyzed based on exosomal protein yield. * indicates significance difference (*n* = 3, *p* < 0.05; unpaired *t*-test).

Exosomes were isolated from SW480 cells using ultracentrifugation. Transmission electron micrographs revealed the rounded structures of exosomes isolated from SW480 cells with and without treatment of FTS (Figure 3a). The particle size of the exosomes was determined using NanoSight. Figure 3b shows the particle size distribution. The average particle sizes of the exosomes derived from the control and FTS-treated SW480 cells were 114.3 ± 12.35 nm and 122.3 ± 8.07 nm, respectively. Western blot analysis (Figure 3c, Figures S2–S5) showed the expression of common exosomal markers (CD63, CD9, CD81 and HSC70) with enrichment of CD9 and CD81 in the isolated exosomes. When quantifying the total proteins in the isolated exosomes using Bradford assay (Figure 3d), a significantly higher amount of exosomal protein (4.23 ± 0.25 µg/10^6^ cells) was isolated from treated cells in comparison to the control group (1.77 ± 0.38 µg/10^6^ cells), indicating that the inhibition of KRAS using FTS enhances the production of exosomes from SW480 cells.

### 3.4. Proteomic Analysis of SW480 Cells-Derived Exosomes

#### 3.4.1. Protein Identification and Network Analysis

To determine the effect of KRAS mutation on the protein contents of exosomes, the proteins in exosomes derived from both FTS-treated and untreated SW480 cells were profiled using Nano-LC-MS/MS. After applying stringent criteria for the processing of the raw data, a total of 435 proteins were identified across all samples, with defined relative expressions between control and treatment. To further investigate the functions of the exosomal proteins identified in FTS-treated and untreated SW480 cells, the protein list was uploaded to STRING-DB, an online tool for the study of functional annotated protein interaction [14]. A protein-protein interaction network was constructed for the 435 uploaded exosomal proteins identified in this study. In this protein interaction network, 431 nodes with 3191 edges were identified with statistically significant connection. Gene Ontology (GO), which is the method functional enrichment analysis, was then conducted to classify the proteins based on the (1) cellular components, (2) molecular functions and (3) biological processes (Table 1, Table 2 and Table 3). The identified proteins were classified as components of extracellular exosomes. In addition to the molecular characterization (Figure 3), GO analysis of the cellular components in this protein profiling approach further verified the exosomal origin of the vesicle samples isolated from the cell culture in our study (Table 1).

#### 3.4.2. Comparative Proteomics

**Figure 4:**
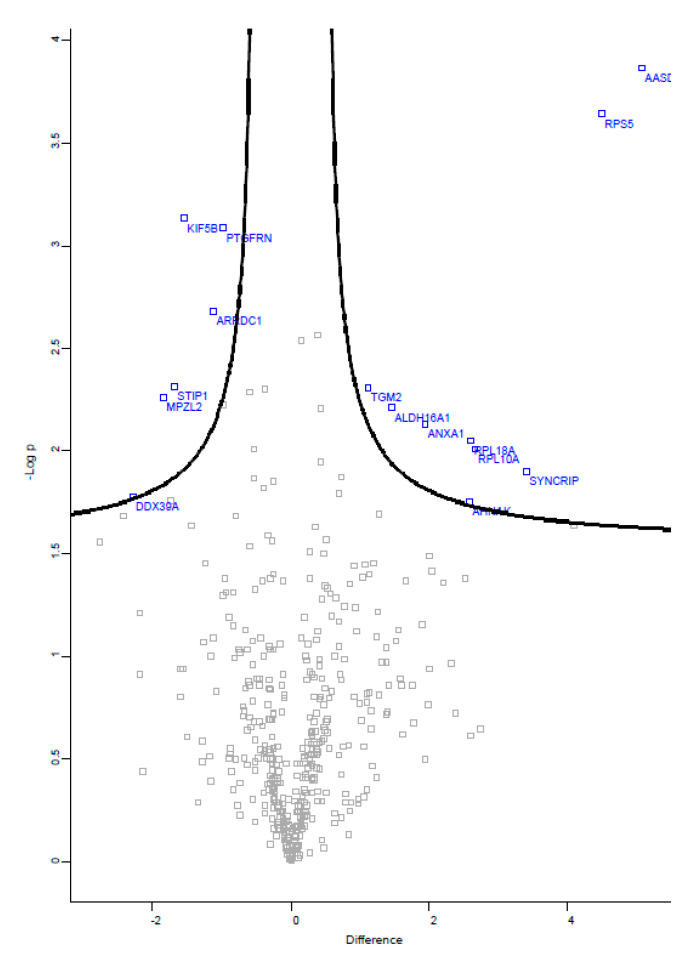
Volcano plot of proteins identified in SW480 cells-derived exosomes. Blue dots are proteins with significant fold changes identified in the exosomes of FTS-treated cells.

Among the 435 proteins that were detected in this study, 15 proteins were found to be significantly regulated by FTS treatment in the SW480 cells-derived exosomes, with equal to or greater than two-fold changes when compared to the control group (Figure 4). The list includes nine up-regulated and six down-regulated proteins identified in the exosomes derived from FTS-treated SW480 cells (Table 4). Among the up-regulated proteins, a remarkably high level (34 times higher) of acyl-coA synthetase family member 4 (ACSL4, product of gene AASDH) was detected as one of the exosomal proteins significantly responsive to FTS treatment.

#### 3.4.3. Verification

**Figure 5:**
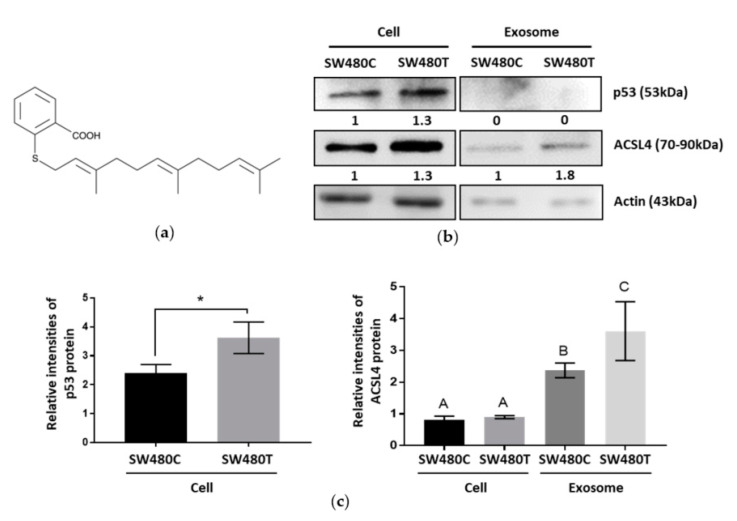
Western blot analysis of the effect of FTS on SW480 cells and the secreted exosomes. (**a**) Structure of FTS compound; (**b**) Western blotting of p53 and ACSL4 with actin as the loading control; (**c**) Relative intensities of p53 and ACSL4 proteins expression which normalized to actin. Means with different letters indicate significant difference between groups (*n* = 3, *p* < 0.05; one-way ANOVA-Tukey’s multiple comparison tests).

Farnesylthiosalicylic acid (FTS) is an S-farnesylcysteine analog, a compound with a benzene ring and a carbon chain (Figure 5a). Network analysis in our proteomic study classified the exosomal proteins secreted by FTS-treated SW480 cells as organic cyclic compound-binding proteins (Table 2), implying the compound may be expelled from the treated cells through exosome secretion. On the other hand, the identified proteins were also found to be part of the cellular stress response pathway (Table 3). Previous studies reported that the tumor suppressor protein p53 played a role in mediating the release of exosomes in response to stress [15]. Western blot analysis shows that treatment with FTS significantly increases p53 in SW480 cells, but not in the exosomes (Figure 5b,c, Figures S6 and S7), indicating that stress was induced in the treated cells. In the proteomic analysis, the inhibition of KRAS using FTS in SW480 cells elevated ACSL4 in the exosomes. The ACSL4 protein plays a role in the survival of cancer cells. The increased level of this protein in exosomes in response to FTS treatment was verified using Western blotting (Figure 5b,c).

### 3.5. Effect of SW480 Cells-Derived Exosomes on Survival of Recipient Cells

#### 3.5.1. Exosome Uptake

**Figure 6:**
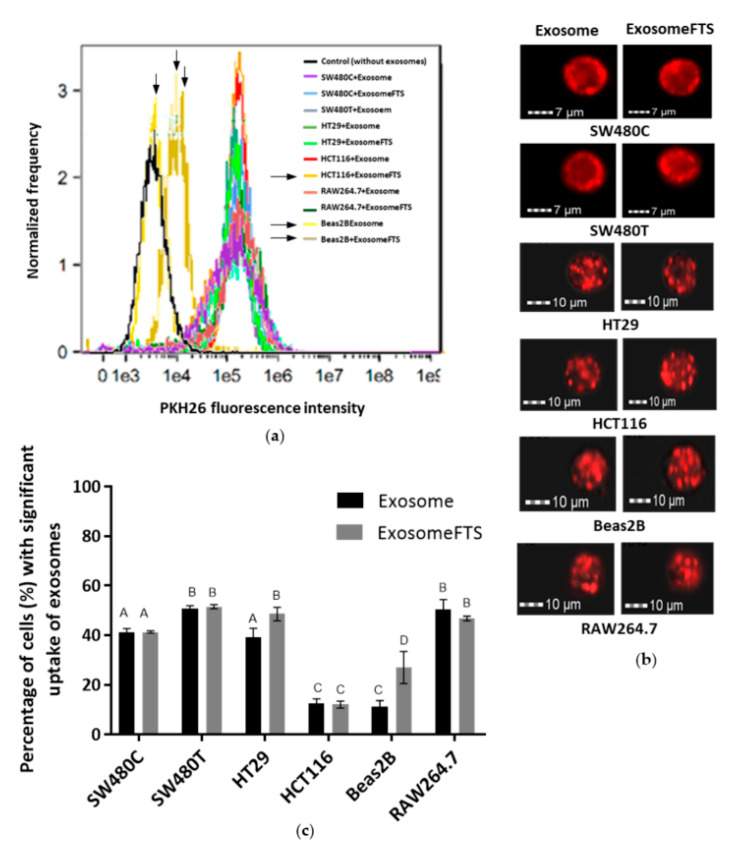
Imaging flow cytometry analysis of various cell types with uptake of exosomes derived from SW480 cells after 24 h of incubation (SW480C—Control cells; SW480T—FTS-treated cells). (**a**) The flow cytometry graphs show red fluorescence intensities detected in various cell types that received PKH26-stained exosomes; (**b**) Representative images of recipient cells captured using Amnis ImageStream X Mark II; (**c**) Percentage of cell uptake with significant quantities of PKH26-stained exosomes based on the spot count analysis using IDEAS software. Means with different letters indicate a significant difference between groups (*n* = 3, *p* < 0.05; one-way ANOVA-Tukey’s multiple comparison tests).

Exosomes were collected from the SW480 cells with and without treatment of FTS and stained using PKH26 fluorescence stain. Six different cell lines, including SW480, were incubated with the stained exosomes, and the uptake was studied using imaging flow cytometry. As a preliminary screening of exosome uptake, the flow cytometry analysis indicated the right shift of the fluorescence intensities detected in all cell lines incubated with PKH26-stained exosomes, when compared to the control cells (without exosome incubation), indicating that all of these cell lines received SW480 cells-derived exosomes. However, Beas2B, as an epithelial cell line, shows remarkably less exosome uptake when compared to other cancer cell lines (Figure 6a). Further, a spot count analysis was performed using IDEAS software to quantify the amount of exosomes taken up by the recipient cells within 24 h, visualized as the red fluorescence spots shown in Figure 6b. The spot count of exosomes can be categorized into ‘low’, ‘medium’ and ‘high’ based on the fluorescence images. The ‘significant’ spot count combined both the statistics generated for both ‘medium’ and ‘high’ spot counts, which indicates the ability of a single cell to take up large amounts of exosomes. ‘Significant’ exosome uptake was quantified for each cell line and shown in Figure 6c.

#### 3.5.2. Viability of Recipient Cells

**Figure 7:**
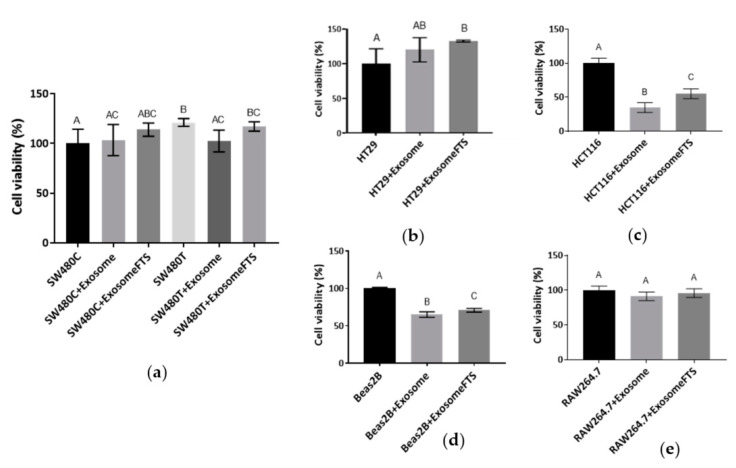
Viability of various cell types with uptake of exosomes derived by SW480 cells after 24 h of incubation (SW480C—Control cells; SW480T—FTS-treated cells; Exosome—Exosomes derived by SW480C; ExosomeFTS—Exosomes derived by SW480T). The viability of cell lines (**a**) SW480, (**b**) HT29, (**c**) HCT116, (**d**) Beas2B and (**e**) RAW264.7 was determined using MTT assay. Means with different letters indicate a significant difference between groups (*n* = 3, *p* < 0.05; one-way ANOVA-Tukey’s multiple comparison tests).

Figure 6c indicates that the significant uptake of exosomes is varied depending on the recipient cell types. At the same time, the survival of the various cell lines after the uptake of exosomes was examined using MTT assay (Figure 7). Treatment with a low dose (50 µM) of FTS increased the viability of SW480 cells (Figure 2a) and the uptake of exosomes from their own cell type (Figure 6c). For the treated cells to receive the exosomes from the control group without treatment, decreased viability of SW480recipient cells was observed after the uptake. However, the exosomes secreted by FTS-treated cells could restore SW480 recipient cells (Figure 7a). For HT29 (CRC cell line that contains wild-type KRAS), RAW264.7 (mouse macrophage-like cells) and SW480 without treatment, more than 40% of the cells from these lines received significant levels of SW480 cells-derived exosomes (Figure 6c), but no significant change of cell viability was observed after the exosome uptake (Figure 7a,b,e). However, the survival of Beas2B and HCT116 (CRC with mutated KRAS G13D), with less acceptance of exosomes from SW480 cells (~10% of the cells with uptake, Figure 6c), was severely attenuated. Intriguingly, exosomes from FTS-treated cells could cause a moderate but significant restoration of survival for these cell lines (Figure 7c,d). In general, the inhibition of KRAS in SW480 cells using FTS induces the secretion of exosomes that could enhance (moderately or significantly) the viability of recipient cells, except for the murine RAW264.7 line.

## 4. Discussion

Exosomes enriched in late endosomes were defined as a type of extracellular vesicle containing the tetraspanins CD63, which had been known as a protein located in MVBs [16]. In our preliminary screening, we found that SW480 cells bearing KRAS G12V mutations expressed the highest level of CD63-GFP, followed by HCT116 (KRAS G13D mutation), while HT29 with wild-type KRAS expressed low levels of CD63-GFP. Therefore, SW480 was selected as the model for our study aiming to investigate the link between KRAS activities and the regulation of exosomes in cancers. The mutation of KRAS is known to cause constitutive the activation of oncogenic signaling pathway [2]. Our initial hypothesis was that the higher activities of mutated KRAS in SW480 may be the cause for the higher levels of exosomes observed when compared to HT29 cells with wild-type KRAS, which are accompanied by low levels of the vesicles. Therefore, the inhibition of mutated KRAS in SW480 cells should reduce exosome production if KRAS plays a role in exosome formation. In this study, farnesylthiosalicylic acid (FTS) was used as the inhibitor to block KRAS activity [11,12]. SW480 remained viable when treated with a sub-lethal dose (50 µM) of FTS that significantly reduced KRAS protein levels in the cells. Unexpectedly, we observed that the inhibition of KRAS by FTS induced higher exosome production from SW480 cells. This finding implies KRAS activation and exosome formation may not be associated through a direct cause-effect relationship.

The protein composition of exosomes has gained great interest, as these nanovesicles are able to transport cellular materials that can potentially induce changes in the recipient cells [17]. The effect of KRAS inhibition by FTS on the proteins that sorted and packed SW480-derived exosomes was examined through a proteomic analysis. The analysis identified a total of 435 exosomal proteins that showed changes of their expression levels in response to FTS treatment. One of the interesting outcomes of the network analysis using STRING is that the identified proteins were connected and mapped on the stress response pathway. Previously, exosomes were reported to be secreted by cells in response to stress, and this process was presumed to be mediated by a tumor suppressor protein, p53 [15]. On the other hand, suppression of KRAS activity could significantly induce the expression of p53 in cells [18]. Consistent with this previous finding, Western blot analysis in our study showed that FTS treatment increased p53 in SW480 cells, implying that KRAS inhibition by FTS may trigger stress in the colon cancer cells, and p53 as a stress sensor [19] could possibly mediate the secretion of more exosomes as a cellular response to the intervention. Intriguingly, our proteomics analysis indicated that 40S ribosomal protein S5 (RPS5) was increased in the exosomes derived from FTS-treated SW480 cells. RPS5 is the major component of the 40S subunit of ribosomes. In a study investigating how p53 may be involved in ribosomal biogenesis, RPS5 was found to contribute to the activation and stabilization of the p53 protein [20]. In this study, p53 was not detected in SW480-derived exosomes, but increased levels of RPS5 in the FTS-induced exosomes may imply a p53-related stress response in colon cancer cells with the inhibition of KRAS activity.

Among all the proteins identified with significant changes in exosomes from FTS-treated cells, the highest fold change in the acyl-coA synthetase family member 4 (AASDH or ACSL4) was observed. A level of ACSL4 up to 34 times higher was identified in the exosomes derived from treated SW480 cells when compared to those from untreated control cells. ACSL4 belongs to a group of enzymes which are involved in lipid synthesis and β-oxidation by converting long chain fatty acids into fatty acyl-CoA esters [21]. It was generally up-regulated in cancer cell types, particularly colon, breast and liver cancers, and originated from tissues with higher lipid metabolic rates [22,23,24]. Interestingly, a high level of acyl-coA synthetase family member 3 (ACSL3) was observed in human lung cancer, and the enzyme was found to play an essential role in the tumorigenesis of lung cancers bearing KRAS G13D mutation [25]. Together, these findings imply the oncogenic property of ACSL4 in colon cancers, with 30–40% of the tumors carrying a KRAS mutation, in which KRAS G12V mutation is one of the major pathologic features of SW480 as a colorectal cancer cell line. Oncoproteins play an important role in regulating the survival of cancer cells. It is interesting to speculate that more ACSL4 was packed in the exosomes of stressed SW480 cells as a surviving signal to protect their neighbors. This speculation is further supported by a study that observed that more exosomes were secreted by mast cells that were exposed to oxidative stress. The stress-induced exosomes were involved in the delivery of protective signals in the form of RNA to reduce cell death in recipient cells [26]. Activated KRAS and its downstream effectors were involved in blocking endoplasmic reticulum (ER) stress-induced cell death in cancers [27]. In this study, we speculate SW480 cells may undergo significant levels of ER stress after their constitutively activated KRAS signaling was inhibited by FTS. The sub-lethal dose of FTS used in this study increased viability and exosome uptake in SW480 cells. A possible explanation would be that these cells were about (but not yet) to reach a critical threshold that would cause the abrupt shift to cell death, and the uptake of more exosomes containing protective signal could possibly be one of the defend mechanisms in SW480 cells to achieve survival.

Imaging flow cytometry in this study demonstrated that the exosome transfers between SW480 cells may involve direct fusion of the exosomal membrane with the recipient cell [17,28]. For the cell types other than SW480, red fluorescence-stained vesicles were observed in recipient cells, implying that exosome uptake may occur via endocytosis [29]. All the recipient cells were exposed to the same inoculum of exosomes (0.5 μg/well). Two out of five tested cell lines (Beas2B and HCT116) show low acceptance of exosomes, but the effect of the uptake on recipient cell viability is significant. The low acceptance implies that exosomes from SW480 cells may induce unfavorable cellular responses in these cell lines. RAW264.7 (macrophage-like cells) was capable of taking up more exosomes, which may due to its phagocytic nature, as phagocytic cells are prone to take up more exosomes when compared to non-phagocytic cells [30]. Exosomes taken up via phagocytosis could be subjected to lysosomal clearance [29], and thus the significant uptake has no impact on the viability of RAW264.7 cells. The mechanisms underlying the changes induced by exosome uptake in recipient cells remain largely unclear [31]. Our observations of the uptake of SW480-derived exosomes by different cell types may provide a preliminary clue to support other studies that found that exosomes secreted by donor cells were non-selective on their recipient cells, but depended on the recipient cells for their uptake mechanism, which can vary between cell types [31].

## 5. Conclusions

FTS (Salirasib) has shown potential as an anti-KRAS drug to be tested in clinical trials [32,33]. However, there were studies that demonstrated that FTS alone promotes cell growth and induces the survival pathway in cancers carrying KRAS mutations [34]. The current study demonstrated that the inhibition of KRAS by FTS stimulated higher exosome secretion from colon cancer cells, possibly as a stress response to the drug intervention. The significantly high levels of ACSL4 detected in these exosomes may serve as the protective signal that promotes the survival of neighboring cancer cells. Together, these findings provide new perspective on the protective role of cancer-derived exosomes, which may aid in the improvement of the therapeutic approach to targeting KRAS mutation in colon cancers.

## Figures and Tables

**Table 1:** Gene Ontology (GO) analysis of cellular components.

Cellular Components (GO)			
Pathway ID	Pathway Description	Count in Gene Set	False Discovery Rate
**GO:0070062**	Extracellular exosomes	319	1.51 × 10^−189^
**GO:0044421**	Extracellular region part	324	4.36 × 10^−158^
**GO:0031988**	Membrane-bound vesicle	316	1.81 × 10^−157^
**GO:0031982**	Vesicle	314	4.01 × 10^−151^
**GO:0005576**	Extracellular region	327	2.3e1 × 10^−138^

Top 5 pathways (with gene set > 300) arranged in sequence starting from pathway with the lowest false discovery rate.

**Table 2:** Gene Ontology (GO) analysis of molecular functions.

Molecular Functions (GO)			
Pathway ID	Pathway Description	Count in Gene Set	False Discovery Rate
**GO:0005515**	Protein binding	236	3.52 × 10^−51^
**GO:0005488**	Binding	317	3.14 × 10^−28^
**GO:0097159**	Organic cyclic compound binding	213	4.42 × 10^−25^
**GO:1901363**	Heterocyclic compound binding	211	5.77 × 10^−25^
**GO:0003674**	Molecular function	299	7.49 × 10^−7^

Top 5 pathways (with gene set > 200) arranged in sequence starting from pathway with the lowest false discovery rate.

**Table 3:** Gene Ontology (GO) analysis of biological processes.

Biological Processes (GO)			
Pathway ID	Pathway Description	Count in Gene Set	False Discovery Rate
**GO:0051234**	Establishment of localization	176	2.52 × 10^−34^
**GO:0006810**	Transport	171	2.94 × 10^−33^
**GO:0051179**	Localization	193	9.41 × 10^−33^
**GO:0006950**	Response to stress	163	6.69 × 10^−29^
**GO:0016043**	Cellular component organization	185	1.18 × 10^−23^

Top 5 pathways (with gene set > 150) arranged in sequence starting from pathway with the lowest false discovery rate.

**Table 4:** Summary of the proteins differentially regulated in exosomes derived from FTS-treated SW480 cells when compared to untreated cells.

Gene ID	Protein Name	Fold Change
*Proteins up-regulated in FTS-treated cells*		
AASDH	Acyl-CoA synthetase family member 4 (ACSL4)	34
RPS5	40S ribosomal protein S5;40S ribosomal protein S5, N-terminally processed	23
SYNCRIP	Heterogeneous nuclear ribonucleoprotein Q	11
RPL10A	60S ribosomal protein L10a	6
RPL18A	60S ribosomal protein L18a	6
AHNAK	Neuroblast differentiation-associated protein AHNAK	6
ANXA1	Annexin A1	4
ALDH16A1	Aldehyde dehydrogenase family 16 member A1	3
TGM2	Protein-glutamine gamma-glutamyltransferase 2	2
*Proteins down-regulated in FTS-treated cells*		
PTGFRN	Prostaglandin F2 receptor negative regulator	2
ARRDC1	Arrestin domain-containing protein 1	2
KIF5B	Kinesin-1 heavy chain	3
STIP1	Stress-induced-phosphoprotein 1	3
MPZL2	Myelin protein zero-like protein 2	4
DDX39A; DDX39B	ATP-dependent RNA helicase DDX39A; Spliceosome RNA helicase DDX39B	5

## Supplementary Materials

The following are available online at https://www.mdpi.com/2079-7737/10/1/58/s1, Figures S1–S7: Replicates of Western blotting analysis.
