# Supplementary material for: Does KRAS Play a Role in the Regulation of Colon Cancer Cells-Derived Exosomes?
Source: Biology (Basel). 2021 Jan 14;10(1):58. doi: 10.3390/biology10010058 (PMC7830101; doi:10.3390/biology10010058)
Supplement: Supplementary file 1 [file biology-10-00058-s001.zip › biology-877935-supplementary.pptx]

## Slide 1
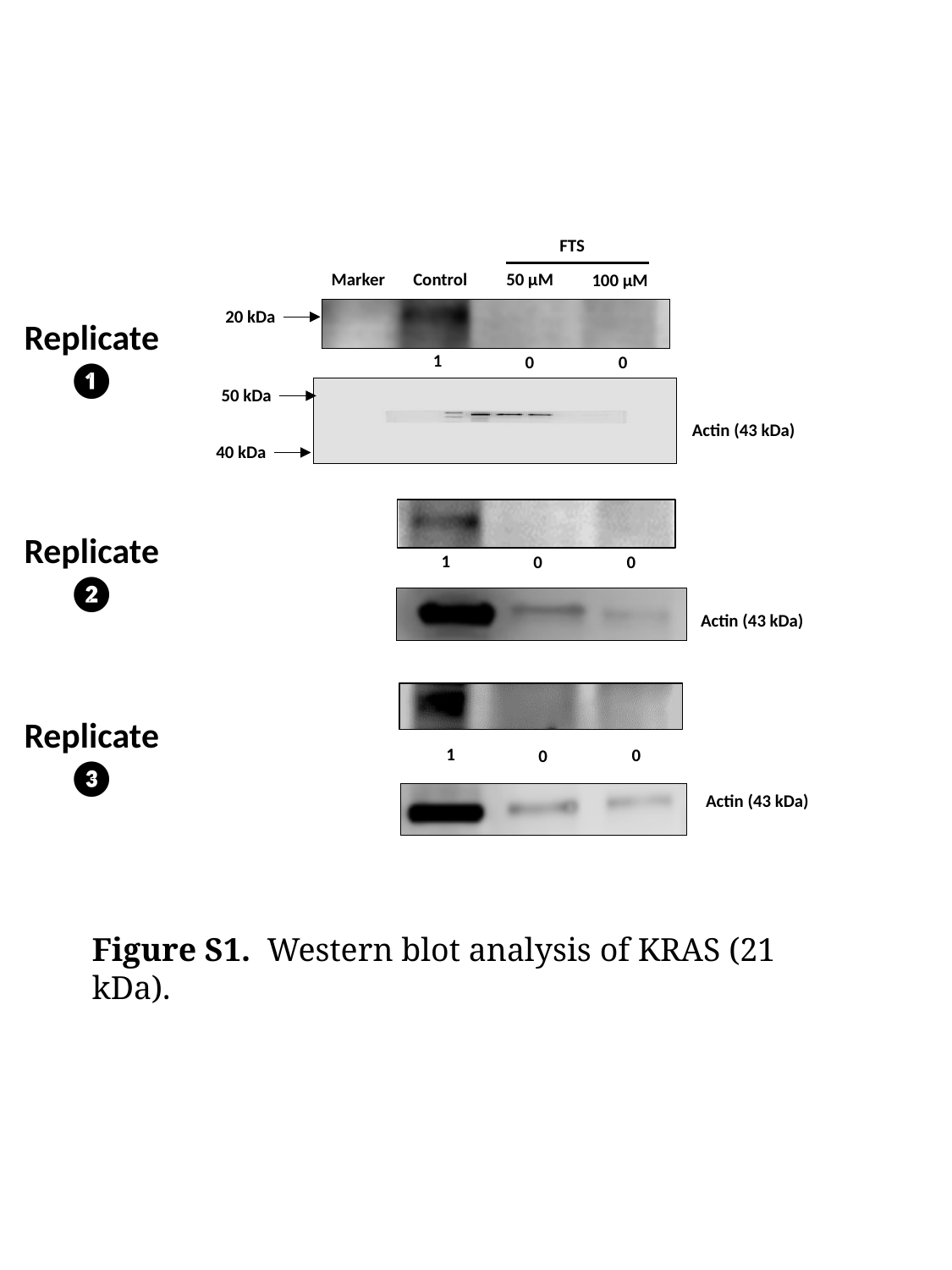

FTS
Marker
50 µM
Control
100 µM
20 kDa
Replicate ❶
1
0
0
50 kDa
Actin (43 kDa)
40 kDa
Replicate ❷
1
0
0
Actin (43 kDa)
Replicate ❸
1
0
0
Actin (43 kDa)
Figure S1.  Western blot analysis of KRAS (21 kDa).

## Slide 2
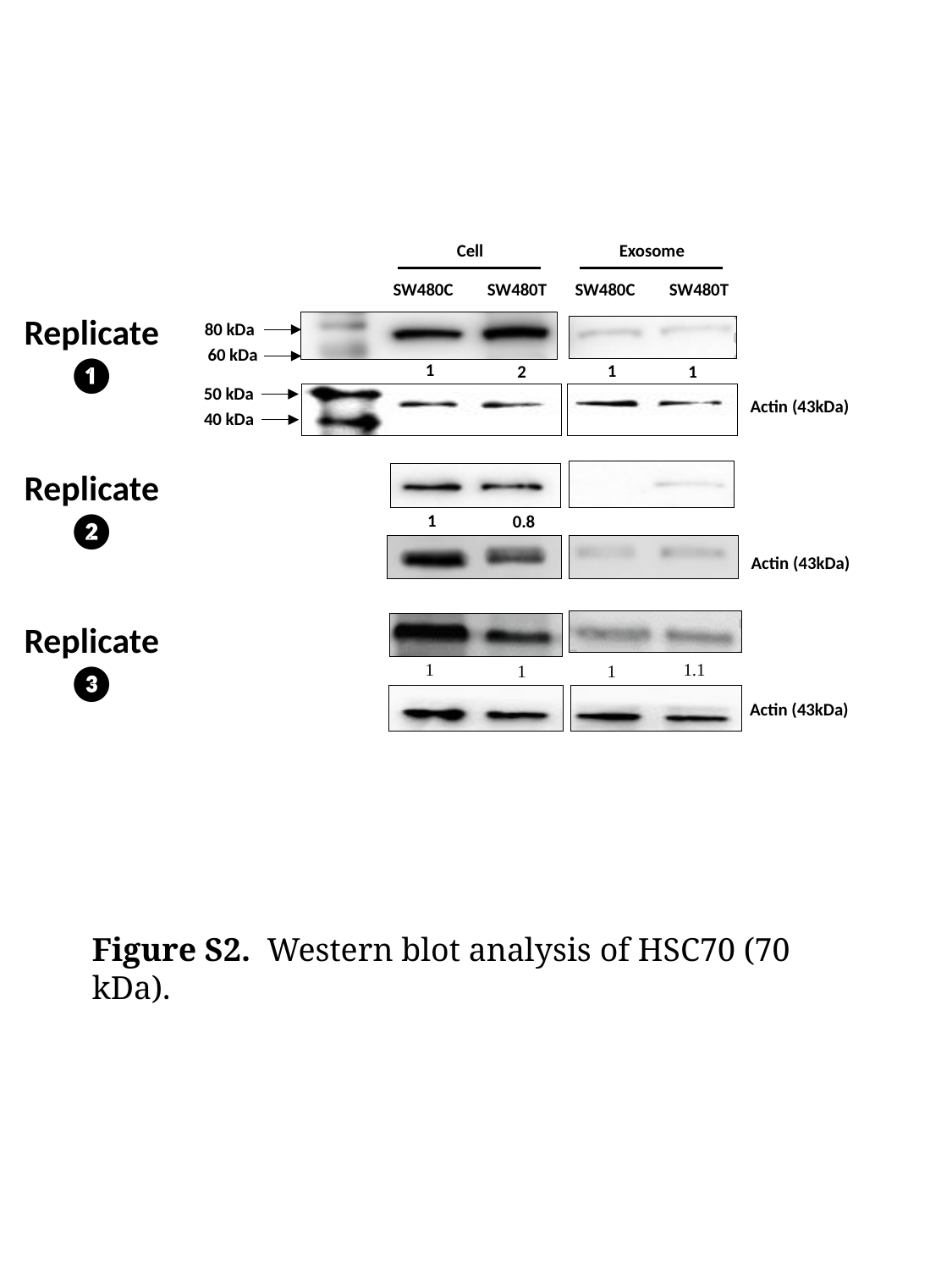

Cell
Exosome
SW480C
SW480C
SW480T
SW480T
Replicate ❶
80 kDa
60 kDa
1
1
1
2
50 kDa
Actin (43kDa)
40 kDa
Replicate ❷
1
0.8
Actin (43kDa)
Replicate ❸
1.1
1
1
1
Actin (43kDa)
Figure S2.  Western blot analysis of HSC70 (70 kDa).

## Slide 3
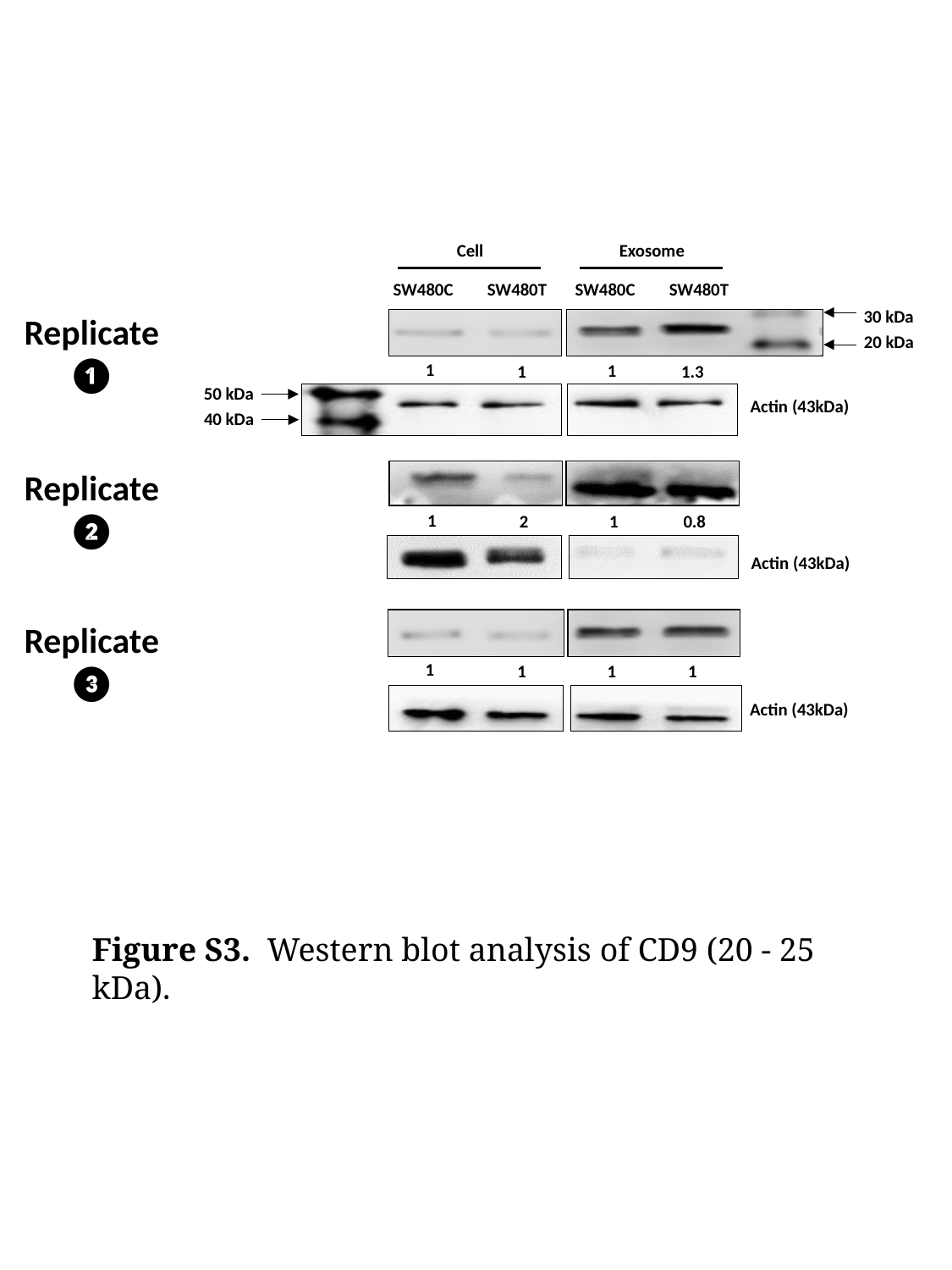

Cell
Exosome
SW480C
SW480C
SW480T
SW480T
30 kDa
Replicate ❶
20 kDa
1
1
1.3
1
50 kDa
Actin (43kDa)
40 kDa
Replicate ❷
1
1
0.8
2
Actin (43kDa)
Replicate ❸
1
1
1
1
Actin (43kDa)
Figure S3.  Western blot analysis of CD9 (20 - 25 kDa).

## Slide 4
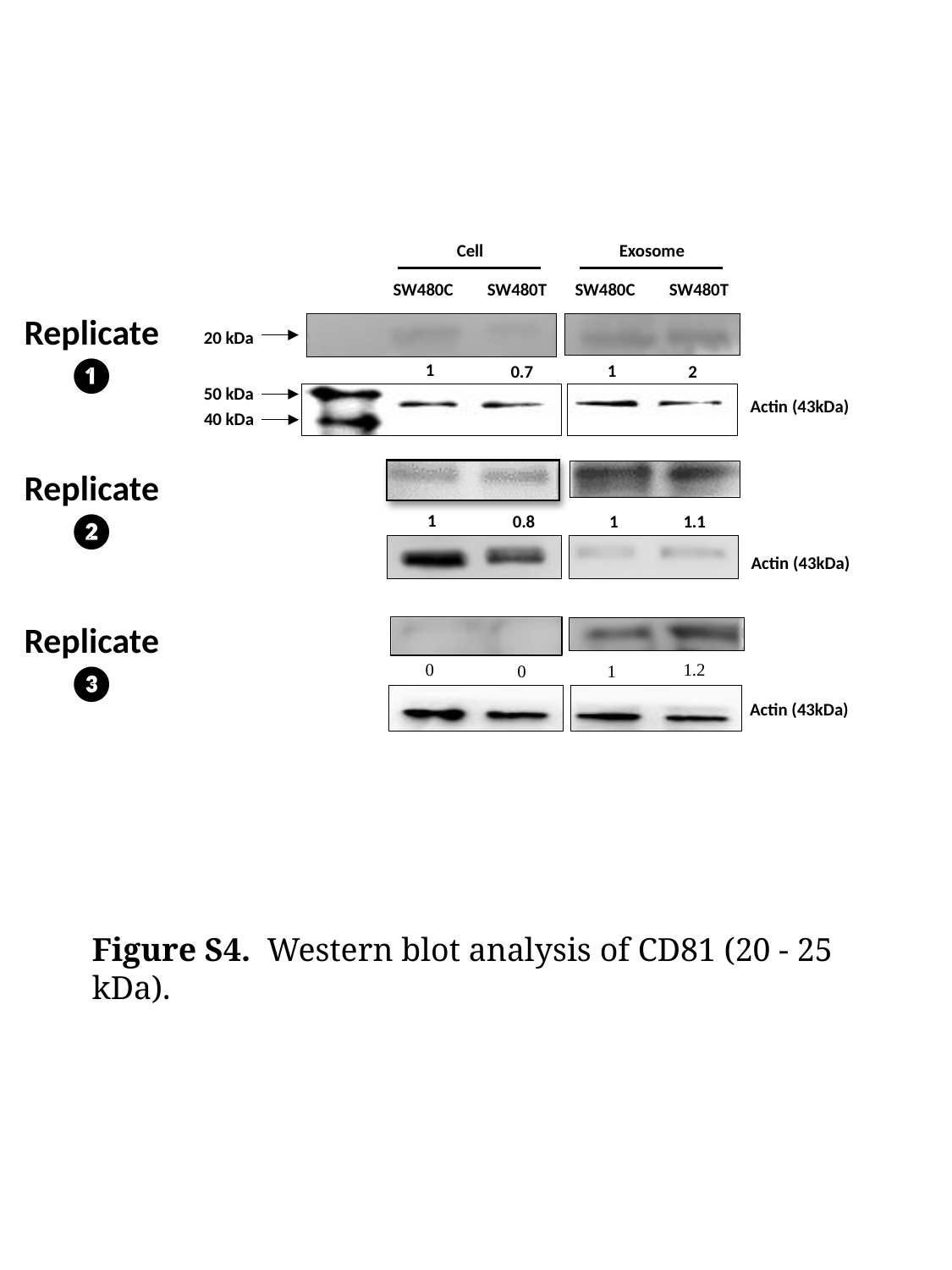

Cell
Exosome
SW480C
SW480C
SW480T
SW480T
Replicate ❶
20 kDa
1
1
2
0.7
50 kDa
Actin (43kDa)
40 kDa
Replicate ❷
1
1
1.1
0.8
Actin (43kDa)
Replicate ❸
1.2
0
1
0
Actin (43kDa)
Figure S4.  Western blot analysis of CD81 (20 - 25 kDa).

## Slide 5
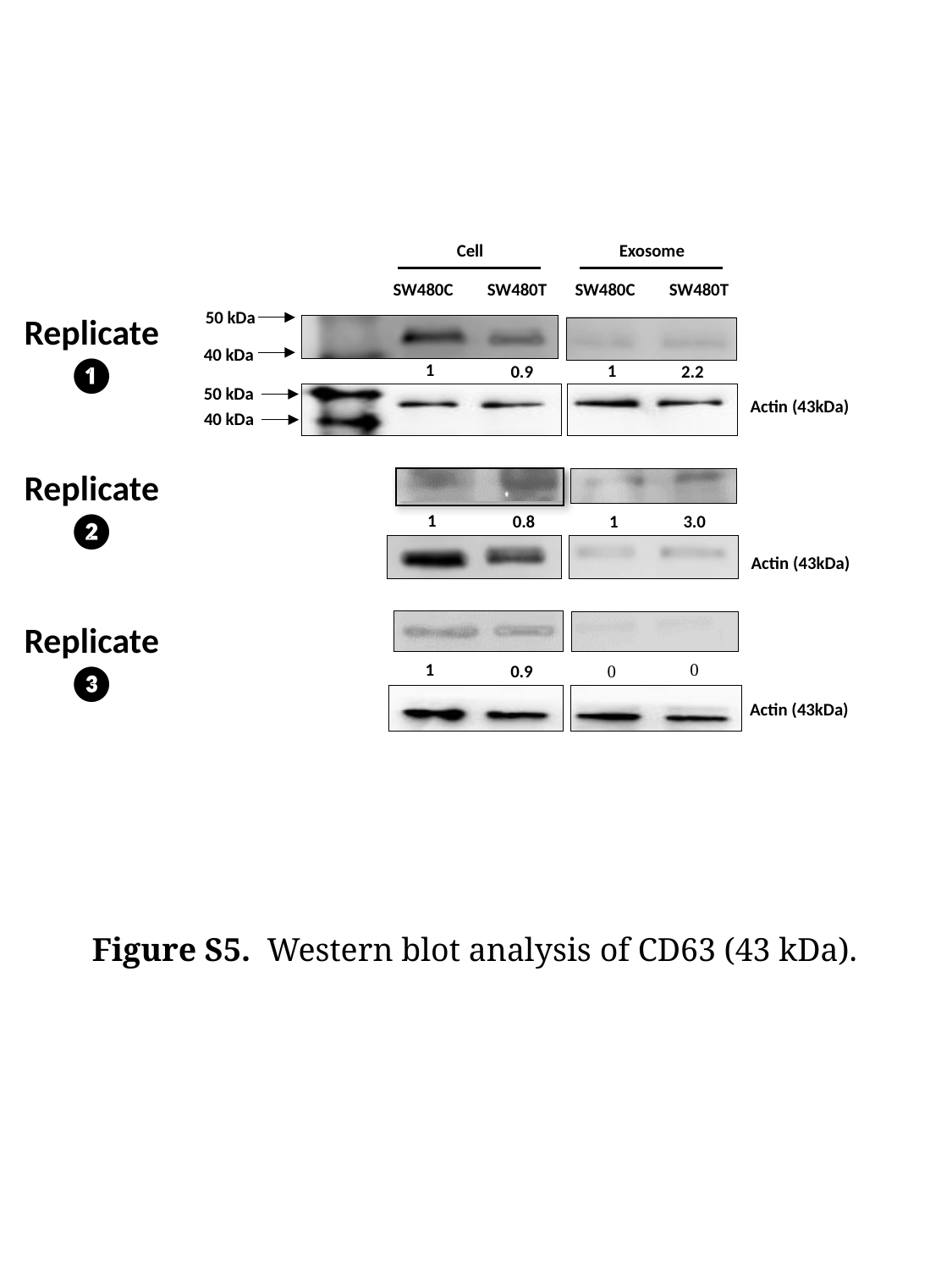

Cell
Exosome
SW480C
SW480C
SW480T
SW480T
50 kDa
Replicate ❶
40 kDa
1
1
2.2
0.9
50 kDa
Actin (43kDa)
40 kDa
Replicate ❷
1
1
3.0
0.8
Actin (43kDa)
Replicate ❸
0
1
0
0.9
Actin (43kDa)
Figure S5.  Western blot analysis of CD63 (43 kDa).

## Slide 6
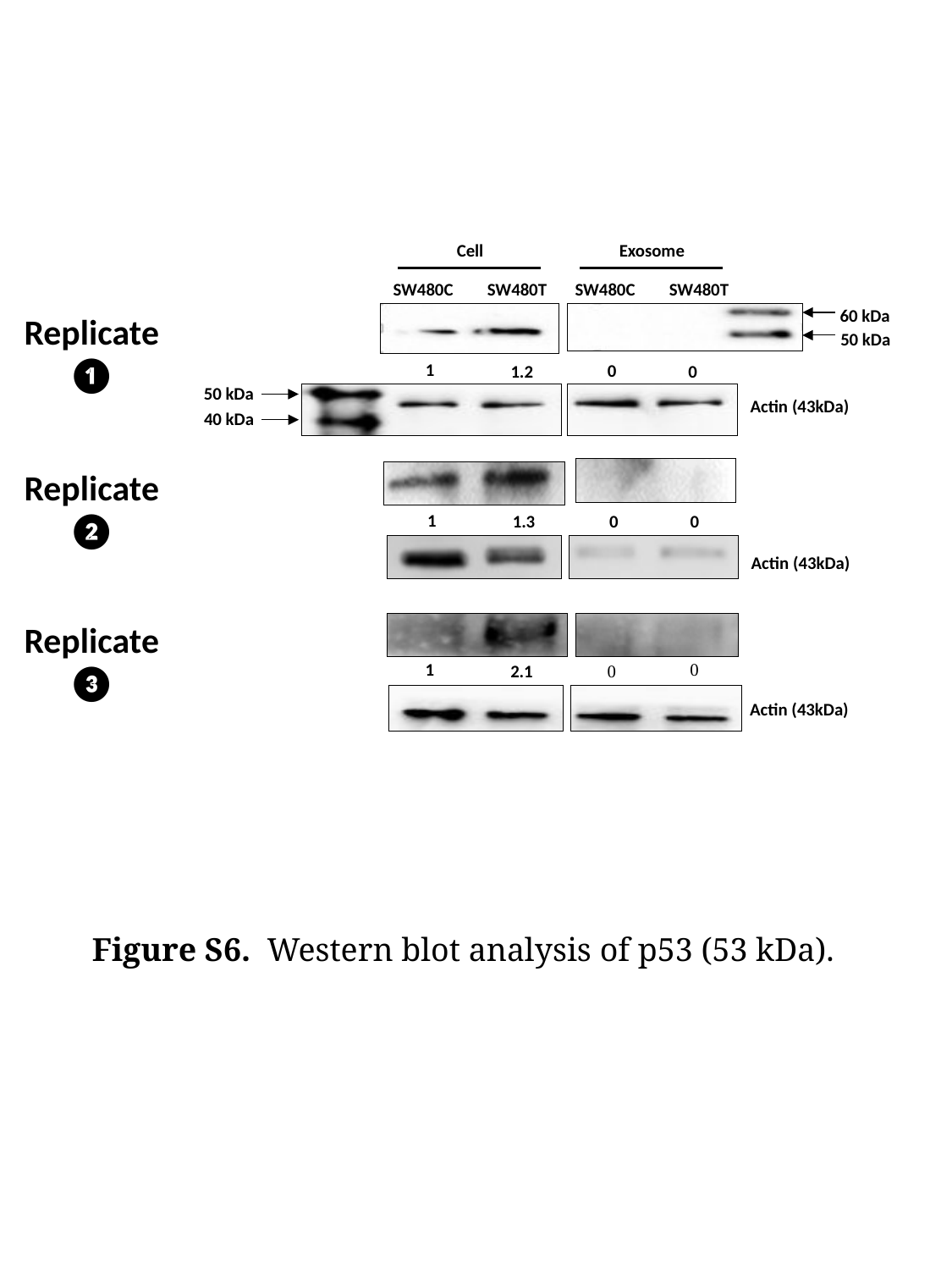

Cell
Exosome
SW480C
SW480C
SW480T
SW480T
60 kDa
Replicate ❶
50 kDa
1
0
0
1.2
50 kDa
Actin (43kDa)
40 kDa
Replicate ❷
1
0
0
1.3
Actin (43kDa)
Replicate ❸
0
1
0
2.1
Actin (43kDa)
Figure S6.  Western blot analysis of p53 (53 kDa).

## Slide 7
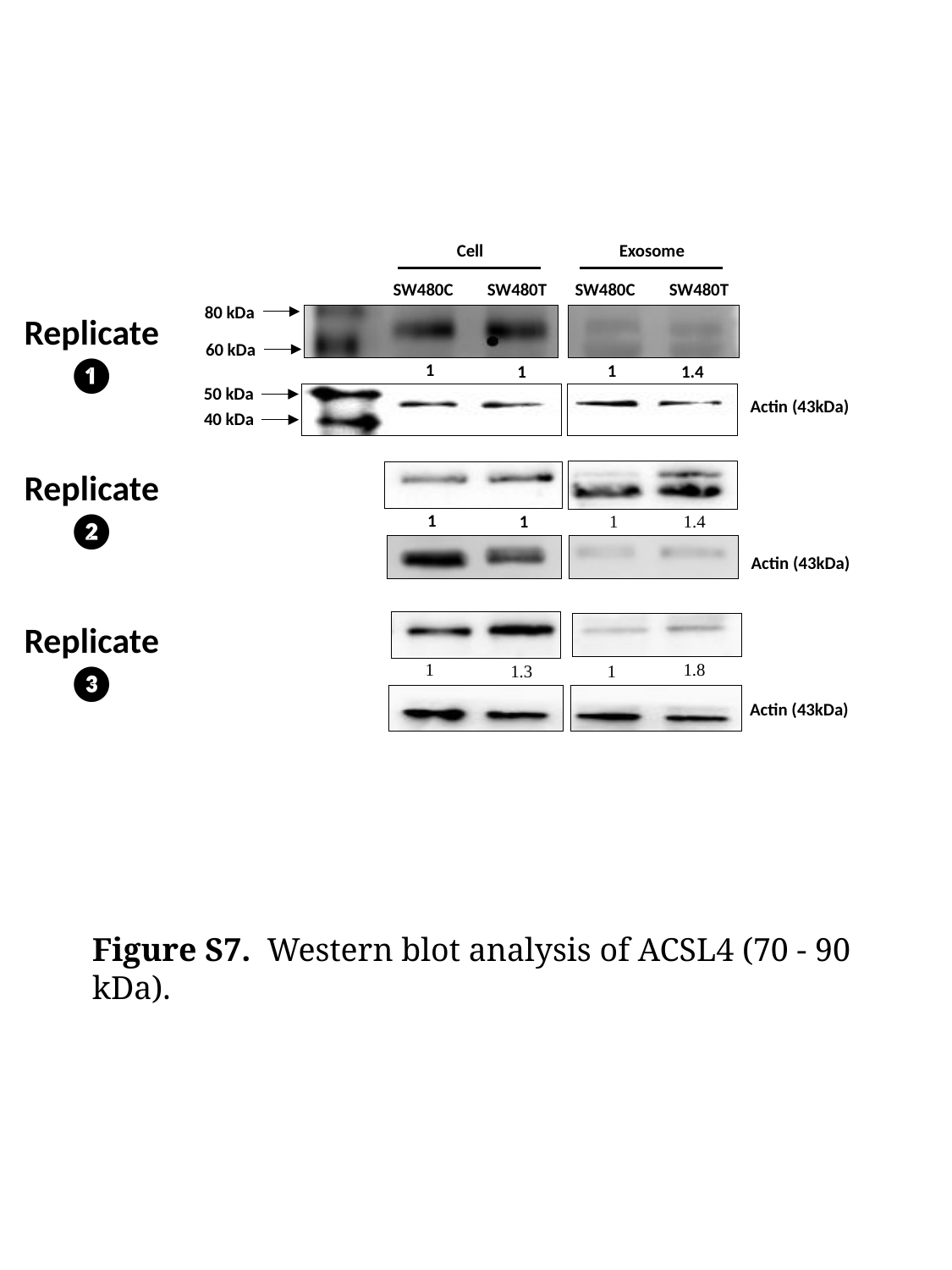

Cell
Exosome
SW480C
SW480C
SW480T
SW480T
80 kDa
Replicate ❶
60 kDa
1
1
1.4
1
50 kDa
Actin (43kDa)
40 kDa
Replicate ❷
1
1
1.4
1
Actin (43kDa)
Replicate ❸
1.8
1
1
1.3
Actin (43kDa)
Figure S7.  Western blot analysis of ACSL4 (70 - 90 kDa).
